# Content validation of the decannulation protocol for adult tracheostomized patients

**DOI:** 10.1590/2317-1782/20232021266en

**Published:** 2023-09-08

**Authors:** Margaret Mendonça Diniz da Côrte, Laélia Cristina Caseiro Vicente, Amélia Augusta de Lima Friche

**Affiliations:** 1 Departamento de Fonoaudiologia, Faculdade de Medicina, Universidade Federal de Minas Gerais - Belo Horizonte (MG), Brasil.

**Keywords:** Data Curation, Validation Study, Tracheostomy, Speech Language Pathologist, Physical Therapists, Physicians

## Abstract

**Purpose:**

Perform content validation of a decannulation protocol for tracheostomized adult patients.

**Methods:**

To validate the content of the protocol developed by speech therapists, the Delphi technique was used. The 11 items of the protocol were judged by experts through rounds via e-mail and were classified as adequate, partially adequate or inadequate, in addition to providing comments and suggestions on each item. 30 speech therapists, 30 respiratory physiotherapists and 30 physicians responsible for the tracheostomy and decannulation procedure were invited. The percentage of agreement adopted was ≥ 80% and the process was interrupted when this percentage was obtained in all items.

**Results:**

At the end of the process, 24 professionals participated in the third round, being 46% speech therapists, 29% physiotherapists and 25% physicians. After the experts' suggestions and comments, two items were kept as they were in the initial protocol, seven were reformulated, six were included and two were excluded. The final version of the protocol included: identification, absence of abundant secretions, characteristics of the secretion, effective cough, ability to remove secretions, tolerate the deflated cuff, aptitude in the decannulation process, level of consciousness, change of cannula to a smaller caliber, absence of current/active infection, spontaneous and effective swallowing of saliva, use of a speech valve, aptitude for occlusion of the cannula, assessment of aptitude for decannulation and objective examinations.

**Conclusion:**

Through the Delphi Technique, the content of the instrument was validated, with substantial changes occurring. The next stage of instrument validation is obtaining evidence of validity in relation to the internal structure.

## INTRODUCTION

Tracheostomy (TT) is a routine hospital surgery procedure, in which a cannula is inserted through a hole in the trachea, communicating it with the outside and making the airway accessible^(1)^.

There has been an increase in hospital TT in the last decade^(1,2)^, estimating that 10 to 15% of patients in intensive care units submitted to mechanical ventilation will need TT as part of their treatment. Besides these, TT is performed in various surgical specialties other than intensive care^(2,3)^.

There are various indications for TT, and when the artificial air passage is no longer needed, the removal process (named decannulation) takes place^(2)^. This is an essential stage in the clinical progress and rehabilitation of patients who have been tracheotomized but do not depend on mechanical ventilation anymore^(4)^. However, there is limited scientific evidence on decannulation and no standardized recommendations or validated protocols for the procedure^(2,3)^.

Data found in the literature regarding the aptitude/readiness for decannulation are limited to experts’ opinions, research studies, single-center experiences, non-validated scores to predict successful decannulation, and some randomized clinical trials focused on organizational issues such as TT teams conducted by intensivists or the effects of specific decisions on outcomes like dysphagia or sleep quality^(2-7)^ - in which decannulation is more often individualized rather than a protocolized process^(6,7)^.

To our knowledge, there is no validated protocol to guide decannulation, as the literature only has some articles on physiological changes that occur after decannulation, with diverging opinions among specialists on the topic^(3,6,7)^.

Validating a protocol is a methodological procedure to assess its quality, which can be defined as the protocol’s capacity to precisely measure that for which it is intended - i.e., the phenomenon in question^(6-8)^. Content validity is the determination of whether the content items are representative based on the judgment of experts in a specific field^(6-8)^.

Content validation makes it possible to associate abstract concepts with observable and measurable indicators addressed by an assessment instrument, determining its representativity, and demonstrating whether it effectively explores the requirements to measure the phenomenon being investigated, through a methodological strategy selected to that end^(1,2,9-13)^.

The Delphi method is widely used in content validation studies and makes up the methodology in various areas and approaches^(9-12)^. A study in the literature used this method and obtained experts’ consensus on a list of TT decannulation prerequisites for adults, as follows: cured or reverted clinical condition that led to TT indication, tolerated TT cannula occlusion without stridor, adequate airway patency (assessed with laryngoscopy), adequate awareness level, intact airway protection laryngopharyngeal functions (coughing, saliva swallowing, capacity to move and eliminate secretions), presence of effective coughing, and absence of new indications for surgery or anesthesia^(10)^. The authors of the said study strongly recommend adding other parameters, such as the type and amount of secretions and frequency of necessary aspiration^(10)^. Thus, the present study deemed it necessary to approach the indications anew with the Delphi method to analyze the variables from an updated perspective, adequate to the reality being researched and including prerequisites absent in the abovementioned study.

The multiple perspectives in a group of experts provide a more valid result than the judgment of a single specialist - even if they are the best specialist in their field^(1,9,12)^.

It must be highlighted that assessment instruments and clinical protocols are integral parts of clinical practice, health assessment, and research, providing scientifically robust results when appropriately developed and validated^(7,14-16)^. Submitting their content to experts’ appraisal refines the instrument for subsequent validation and reliability procedures.

Given the above, this article aimed to validate the content of a multidisciplinary decannulation protocol for tracheotomized adults, using the Delphi method.

## METHODS

The study met the human research ethics criteria, according to Resolution 466/2012 of the National Health Council, and was approved by the institution’s Research Ethics Committee, under approval number 4.458.519. Participating signed an informed consent form, thus agreeing to the procedure and disclosure of the research and its results.

The first decannulation protocol version was initially developed based on a national and international literature review concerning decannulation criteria and data on the medical records of 189 hospitalized tracheotomized adults, which were collected and statistically treated^(17)^. This research conducted the methodological quantitative and qualitative validation study of a temporary TT decannulation protocol.

The initial protocol items approached statistically significant variables in the cited study, adding items considered relevant in the literature in the area^(3-7,17)^. Hence, the first protocol version included the following items: the capacity to remove secretions by swallowing or spitting them; absence of abundant secretions, requiring tracheal tube aspiration three times every 8 hours at the most; tolerance to TT cannula occlusion for at least 48 hours; awareness level scoring 12 to 15 on the Glasgow Coma Scale (GCS); absence of active infections; the presence of spontaneous saliva swallowing; negative blue-dye test result; tolerance to permanently deflated cuff for at least 24 hours; plastic cannula switched for a metal one; absence of dysphagia; oral diet allowed in meals; and use of the speaking valve.

The Delphi method was used to validate the content of the first version of the adult decannulation protocol, collecting experts’ opinions on the topic, tabulating data, and assessing procedure criteria.

The Delphi method is named after the Oracle of Delphi, where ancient Greeks sought counsel and answer about the future^(9)^. It is a research and instrument validation methodological strategy, seeking opinion consensus from a group of specialists, using structured questionnaires organized in phases, cycles, or rounds^(18-20)^. It aims to obtain the maximum consensus from a group of specialists on a given topic when a unanimous opinion is inexistent due to contradictory information or the lack of scientific evidence^(8,21-23)^.

Researchers selected the specialists based on their knowledge and experience on the research topic. They were invited to give their opinion on this specific subject by filling out an assessment questionnaire anonymously^(5,6,21,23)^.

The researchers analyzed the results between each round of questionnaires. They observed the tendencies and diverging opinions along with their justifications, systematizing and compiling them to resend to the group afterward. Thus, after learning the other members’ opinions and the group’s responses, participants had the opportunity to refine, change, or defend their answers and resend them to the researchers to redevelop the questionnaire according to the new information. This process was repeated until they reached a consensus^(24)^.

It was defined that the study sample should comprise at least 30 specialists, experts on the topic, with a specialization, at least 5 years of practical/clinical experience in decannulating tracheotomized patients, and distinct academic training (10 physicians responsible for TT and decannulation procedures, 10 respiratory physical therapists, and 10 speech-language pathologists).

Throughout the Delphi method, a 30 to 50% abstention rate is expected in the first round, and 20 to 30% in the second one^(24)^. Hence, 90 participants were invited (30 SLH therapists, 30 respiratory physical therapists, and 30 physicians responsible for TT and decannulation procedures).

Specialists were invited via e-mail, which formally presented the study objectives, purpose, development, stages, estimated time, deadlines to return questionnaires, and other details inherent to the study. A protocol explanation handbook and a link to the online protocol assessment questionnaire were annexed to the e-mail.

They scored all items in the initial protocol - as well as in the reformulated protocol based on the specialists’ suggestions, which was resent for appreciation - using a Likert scale, as adequate (3), partially adequate (2), or inadequate (1).

The specialists’ observations, comments, and suggestions were recorded in an Excel spreadsheet regarding each item they assessed for later analysis and changes.

In each round, the agreement between specialists’ appraisals was assessed with the content validity index (CVI)^(11,12,25)^, calculated by dividing the number of assessors who agreed with the item by the total number of assessors. Specialists’ observations and suggestions were recorded in a separate file and used in each round to reformulate and adjust the protocol items.

The percentage of agreement used in each round to select variables considered appropriate to the protocol - chosen according to indications in the literature - was 80% or above^(6,19)^. Items were kept when their CVI was ≥ 80% and revised when their reformulation had been suggested. Those whose CVI was ≤ 80% were excluded. After adjustments had been made according to the specialists’ suggestions, the protocol was resent to them for a new appraisal. The process was concluded when all protocol items reached the percentage of agreement.

Participants were characterized regarding their profession/occupation and sociodemographic characteristics, such as age, sex, occupation, time since graduation, postgraduate degree, and time of experience with tracheotomized patients. Hence, descriptive analysis was performed with absolute and relative frequencies.

## RESULTS

**Figure 1:**
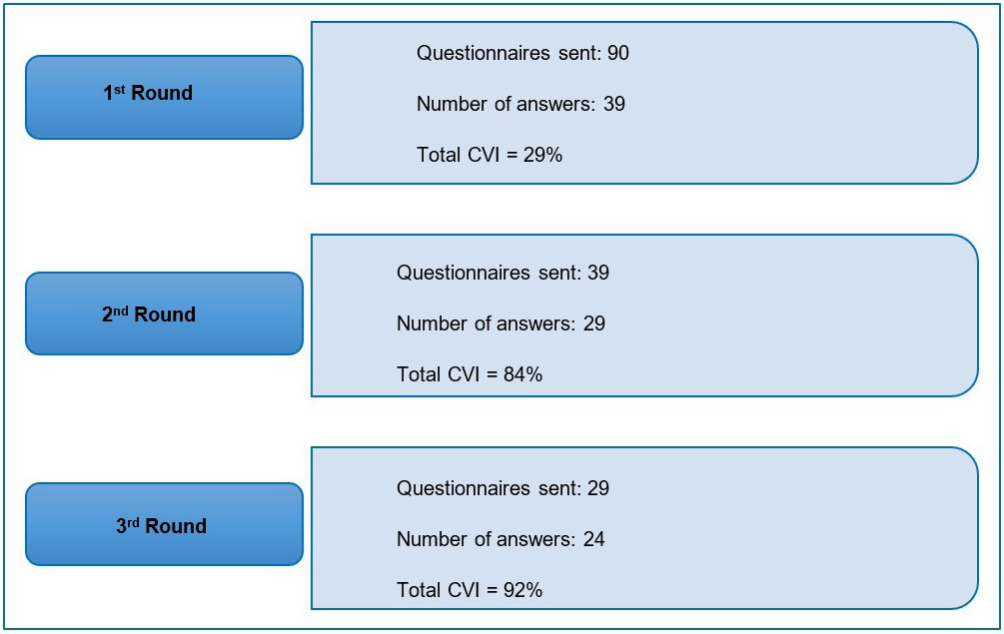
Arrangement of the three Delphi method rounds

Protocol content validation needed three rounds until all its items reached an 80% agreement between specialists (Figure 1).

In the first round, 39 of the 90 invited specialists answered the questionnaire - 19 SLH therapists, 11 physicians, and nine physical therapists. Hence, there was a 57% absence rate from the invitation to the first round. The subsequent absence rates were 26% in the second round (29 out of the 39 specialists answered the second questionnaire) and 17% in the third and last round (24 out of the 29 specialists answered it).

The professionals who participated in the three assessment rounds conducted in this study were 28 to 52 years old, with a mean age of 40 years (SD = 6). The largest number of specialists was that of SLH therapists in the first (49%), second (48%), and third rounds (46%), followed by physicians (28%) and physical therapists (23%) in the first round. In the second and third rounds, the answers were sent by SLH therapists (45.8%), followed by physical therapists (29.2%) and physicians (25%) (Table 1).

**Table 1:** Sociodemographic and professional variables of the judges participating in the Delphi method

**Variable**	% or mean*	
	**First round (n = 39)**	Last round** **(n = 24)**
**Age** (28 to 52 years)	40*	40*; SD = 6***
**Sex**		
Females	52	58
Males	48	42
**Profession**		
Speech-language pathologist	49	46
Physician	28	25
Physical therapist	23	29
Time since graduation		
6 to 10 years	18	21
11 to 20 years	59	54
21 to 30 years	23	25
**Postgraduate degree**		
Specialization	100	100
Master’s	67	29
PhD	62	75
Postdoctoral	28	17
**Time of experience with tracheotomized patients**		
Up to 10 years	31	38
11 to 20 years	54	54
More than 20 years	15	8

* Result presented in mean values **Third round ***Standard deviation

Concerning occupational data, most participants had graduated more than 11 years before (75%), and all of them (100%) had a specialization in their field. In the first round, there was a similar proportion of participants with a master’s (67%) and a doctoral degree (62%), though different from those with a postdoctoral degree (28%). In the third and last round, there was an important difference between the number of professionals with a master’s (29%) and a doctoral degree (75%). The specialists’ predominating time of experience with tracheotomized patients was from 11 to 20 years (Table 1).

After this round, all items reached an ≥ 80% agreement index, thus ending the Delphi method. Hence, the protocol content was validated. The protocol items assessed by specialists are described below (Table 2).

**Table 2:** Items assessed in the three Delphi method rounds

Items assessed	Round/CVI (%)		
	1^st^	2^nd^	3^rd^
Identification data	38*	79*	96
Complementary data	**	96	96
**Aptitude to begin the decannulation process**			
Absence of abundant secretions	59*	90	90
Amount of secretion in each aspiration	**	76*	88
Aspect of the secretion	**	82	88
Capacity to remove secretions	85*	97	97
Effective coughing	64*	90	90
The patient tolerates deflated cuff for 24 hours	**	86	86
**Result:** Apt to begin the process	79*	80	89
**Aptitude for Tracheostomy Cannula Occlusion**			
Awareness level: GCS 9 to 12/13 to l5	59*	83	83
Exchange for a thinner metal cannula	**	83	90
Absence of active infections	59*	90	90
Presence of spontaneous saliva swallowing	95	93	93
Blue-dye test	34*	***	***
Oral diet allowed	59*	***	***
**Result:** Apt for occlusion	66*	83	83
**Aptitude for Decannulation**			
Date/hour of occlusion; plunger, gauze, surgical tape	69*	***	***
Cannula occlusion: Date/hour; Standard: plunger	**	86	86
Occlusion (hours): 24-36/48-72/Not tolerated	79*	83	83
Bronchoscopy: N/S; Date and result	54*	***	***
Objective examinations: Bronchoscopy/Nasolaryngoscopy/Videofluoroscopy	**	93	93
**RESULT**			
Apt for decannulation	74***	***	***
Apt: Tolerated 24 hours. If they were examined: Not contraindicated	**	80*	92
Decannulated: Yes/No; Reason	97	97	96
Date/hour of decannulation and name of the professional	97	93	96

* Reformulated **Included ***Excluded

**Caption:** CVI = Content validity index

The items were maintained unaltered after they reached the ≥ 80% CVI and specialists had no more suggestions for changes - otherwise, they would have been reformulated and resent for a new round of appraisal. Based on the experts’ suggestions and comments, two items were kept as they were from the initial protocol, seven were reformulated, six were included, and two were excluded. The final protocol, after the third round, is available in Appendix A in this article.

According to the experts’ suggestions and comments, the items’ capacity to remove secretions by swallowing or spitting them and the presence of spontaneous saliva swallowing were maintained as they were in the initial protocol.

The seven reformulated items are presented below, along with the experts’ main comments/suggestions: identification data (the participating patients should be identified only with their initials to preserve their identity; the date of admission to the hospital should be included, as well as the patient’s weight and height), absence of abundant secretions (take note of the number of times aspiration had to be made, because there is a difference in having zero or three aspirations; it is essential to quantify aspirated secretion; being able to eliminate secretions is necessary to protect the airways), coughing (not all services have a cough flow meter), awareness level (limit the result to 9 to 15 on GCS, because that profile of patients is more indicated for decannulation), absence of active infections (specify active pulmonary or laryngopharyngeal infections, as pulmonary and neurological changes, for instance, can interfere with decannulation); cannula occlusion; and evaluation of the aptitude for decannulation.

The six other items that were included addressed complementary data, characteristics of the secretion, toleration to deflated cuff, exchange for a thinner cannula, use of the speaking valve, and objective examinations performed.

They suggested excluding the items on the blue dye test and oral diet. The reason why they would have the blue-dye test item removed is that it can be a false negative in up to 50% of the cases and, therefore, is not a reliable parameter to be considered; moreover, SLH clinical assessments have the final word over the test. As for the oral diet, the specialists’ main observations were that the possibility of decannulating a patient is not always related to whether they are apt for an oral diet - analyzed alone, it is not a parameter that indicates criteria and risk for TT decannulation. Thus, there is no relationship between allowing an oral diet and decannulating, which, consequently, is not directly dependent on the former. Furthermore, patients with dysphagia, who cannot and are not allowed to have an oral diet, may have unobstructed airways and the capacity to protect the lower airways, enabling decannulation.

The final protocol, after the third round, is available in Appendix A, in this article.

Lastly, the comparative observation between contents in the initial and final protocols shows the following main changes, based on the specialists’ assessments and observations: between the first and second rounds, the following items were included: interdisciplinary protocol; previous respiratory diseases and dysphagia; reason for TT and complications; TT cannula diameter and absence or presence of a cuff; viscosity characteristics and aspect of the secretion; use of the speaking valve; and tolerance to deflated cuff. Also, the items’ absence of abundant secretions and coughing were modified/reformulated.

Changes between the second and third rounds referred to the amount and aspect of aspirated secretions and the evaluation of coughing.

After the three rounds with the specialists’ suggestions, the following items remained in the final protocol version: identification, absence of abundant secretions, characteristics of the secretion, effective coughing, capacity to remove secretions, tolerance to deflated cuff, criteria: being apt for decannulation, awareness level (GCS), exchange for a thinner cannula, absence of current/active infections, spontaneous effective saliva swallowing, use of the speaking valve, criteria: being apt for cannula occlusion, evaluation of the aptitude for decannulation, objective examinations (Appendix A).

## DISCUSSION

The three participating professional categories are closely related to decannulation, and their decisions determine the conduction and outcomes of the whole process. This enabled a more reliable construction, without individual dominance over the assessment instrument proposed in this study^(1)^.

The lack of validated decannulation protocols in hospitals may lead to clinical and respiratory complications, such as premature decannulation, respiratory failure, secretion accumulated in pharyngeal recess with increased risk of bronchoaspiration, impaired lower airway protection mechanism, lower-airway stridor, sepses, enlarged stoma, and changes in the mucosa^(26)^.

Validating health protocols is an important task to ensure safety, evidence, and quality in actions related to the assistance to patients, especially in hospitals, to promote safe, effective, and efficient actions^(23-26)^.

The Delphi method is used to generate a sample of specialists’ opinions, preventing overassertive individuals from dominating the process. Hence, it has been considered an adequate means of extracting useful data from personal experiences that can be transformed into empirical data for future studies^(7-9)^. Guidelines are developed based on responses to the Delphi method to provide an important base to produce and assess studies and publications^(1,9)^.

Evidence obtained from committees of experts’ reports or opinions and/or respected authorities’ clinical experiences belongs to Level IV in the pyramid of evidence^(22)^. Most specialists agree that the higher the study design is located in the hierarchy, the more rigorous its methodology will be^(22)^.

The specialists who participated in this study had a satisfactory profile of clinical experience in the area, with many years of experience with tracheotomized patients. This corroborates the literature, which states that participating experts must have an affinity with the proposition that is meant to be validated - hence, they must have academic or scientific productions and/or professional experience in the area in which the study is grounded, thus being characterized as experts^(23)^.

All specialists had a specialization postgraduate degree, and a significant number of them had master’s and doctoral degrees. The literature states that good-quality assessments require a panel of experts qualified on the topic, with academic training and expertise appropriate to the issue being analyzed, based on the quality of their contribution^(10,23)^. Expertise refers to a continuum that includes subjective and objective expertise, both related to academic training and experience on the research topic^(23)^. Hence, specialists must be recruited according to their experience and credibility on the topic^(25,26)^. They should be at least 10 (fewer than this does not generate enough ideas) and at the most 50 (larger samples leads to cost inefficiency regarding time, product, and iteration) participating specialists, experts on the topic, with different academic training to broaden the clinical reasoning around the issue at hand^(25-28)^.

The initial proposal in this study was to count on at least 30 participating specialists. However, the final number of participants was 24 in the third round, with a good CVI. Moreover, even though the final number of specialists was smaller than expected, it is still within the suggested in the literature as adequate to maintain the quality of the Delphi method in the consensus of opinions^(1,24,27,29)^.

Abstentions did not change the quality of the content validation process for the decannulation protocol. The lack of homogeneity between professional categories in all rounds may have been a fragility of this study, as the protocol is meant to be multiprofessional.

On the other hand, the analysis of the professionals’ profiles showed that participants have adequate training and time of experience in the decannulation of tracheotomized patients, which enables adequate and appropriate analyses, observations, and suggestions.

Thus, the main changes in the first protocol version according to the specialists’ observations and suggestions refer to the following items: complementary data (specialists suggested including some items after the identification to provide further details on the clinical case), quantification of secretions and identification of their characteristics, tolerance to deflated cuff, use of the speaking valve, standardization of the cannula occlusion resource, and removal of the items on the blue-dye test and oral diet. These changes led to the second version, whose main suggested changes referred to the following items: detailed aspects of the secretions, use of the speaking valve, objective examinations performed, and criteria for the aptitude for decannulation. Changes made in the second round led to the third protocol version, which is available in Appendix A.

Concerning the item on patients being allowed to have an oral diet, an important percentage of participating physicians, physical therapists, and SLH therapists questioned the direct relationship between the patient’s readiness to receive an oral diet and their aptitude for decannulation. No studies were found addressing the relationship between being decannulated and receiving an oral diet.

Among the specialists’ considerations, they suggested excluding or disregarding this item as an important part of the protocol. Most of them were SLH therapists, who “are the professionals legally certified to assess, diagnose, and provide SLH treatment of oropharyngeal dysphagia and manage it in newborns, children, adolescents, adults, and older adults” (Federal SLH Council Resolution no. 356, of December 6, 2008). Therefore, it was decided to exclude this item from the protocol.

The decannulation protocol proposed and assessed in the study^(16)^ was developed by surveying important data in the literature regarding decannulation. It considered clinical and statistical criteria, and its content was substantially modified and adjusted based on the specialists’ assessments. Thus, the authors considered the protocol validated regarding its content. As described in the literature, content validity determines if content items are representative, based on the judgment of specialists in a specific area, defining whether the protocol’s content effectively explores the requirements to measure a certain phenomenon to be investigated^(1,2)^ - which was the process that took place in this validation study.

Despite the possible abovementioned limitations, this study is to our knowledge the first one to propose the validation of a decannulation protocol from a multiprofessional perspective. The final protocol encompassed the most important items for decannulation, helping identify clinical and respiratory characteristics and, consequently, correct decision-making to prevent complications in this process.

Future protocol validation and reliability stages must take place in different services, applying it to hospitalized patients.

## CONCLUSION

This study described the validation, with the Delphi method, of a multidisciplinary decannulation protocol for tracheotomized adults. Given the results, the validity evidence was considered satisfactory.

The specialists’ contributions helped improved the instrument and validated its content. The next validation stage is to obtain validity evidence regarding its internal structure, and then submit the instrument to other reliability and validation parameters, by applying the protocol to the target population.

## Appendix A Final protocol

**Tracheostomy Decannulation Protocol**

Instructions: Interdisciplinary protocol: physicians, physical therapists, and speech-language pathologists. Evaluations and decisions on the decannulation process must be made by the team.

Evaluation date: Professional in charge:

Identification data:

Name (Only number identification for the hospital data collection):

DOB: Age: years Sex: F () M () Weight: Kg Height: meters

Complementary Data for Initial Assessment:

Date of admission: Diagnosis at admission:

Comorbidities/Progressive respiratory diseases/Previous dysphagia:

Tracheostomy: Date: Reason:

Procedure complications: No () Yes () Please, describe:

Cannula: Plastic/Silicone: with an inner cannula () without an inner cannula () Metal ()

Cuff: Absent () Inflated: Yes () No () Cannula diameter (number):

**APTITUDE TO BEGIN THE DECANNULATION PROCESS**

1. Absence of abundant secretions: ≤ 3 tube aspirations within 24 hours: Yes () No ()

Instructions:

a) consider the number of tube aspirations per day (24 hours) performed by the nursing, physical therapy, and speech-language pathologist teams

b) consider the amount of aspirated secretion (in the aspirations overall): Small: the secretion reached only the aspiration tube; Moderate: it reached the beginning of the extension of the vacuum; Large: it reached the aspiration container.

2. Characteristics of the secretion:

a) Viscosity: Thin () Thick ()

b) Aspect: Hyaline: clear, transparent () Purulent () Mucopurulent () Sanguinolent () Sanguinopurulent ()

3. Effective spontaneous and/or voluntary coughing to move secretions from the airways:

Yes () No ()

4. Capacity to remove secretions by swallowing or spitting: Yes () No ()

5. Tolerates deflated cuff for 24 hours: Yes () No ()

Note: The patient remains clinically stable and has an adequate breathing pattern with the cuff permanently deflated for 24 hours of the day

Criteria for the aptitude to begin the decannulation process:

All items with a “yes” answer and item 3 with “thin” and “hyaline” answers

**Result:** Apt to begin the decannulation process: Yes () No ()

**APTITUDE FOR TRACHEOSTOMY CANNULA OCCLUSION**

6. Awareness level (Glasgow Coma Scale) score 9 to 15: Yes () No ()

7. Cannula exchanged for a thinner one: Yes () No ()

8. Absence of current/active infections (Criteria: antibiotic therapy, presence of leukocytosis): Yes () No ()

**Consider:** Active pulmonary or laryngopharyngeal infections; sepsis; infections with delirium episodes.

9. Presence of spontaneous and effective saliva swallowing: Yes () No ()

10. Use of the speaking valve: Yes () No ()

**Note:** The patient must be clinically stable and have an adequate breathing pattern, constantly using the speaking valve. This complementary information is meant for use in services that provide speaking valves.

Criteria for the aptitude for cannula occlusion:

Ideal: All items with a “yes” answer. Safely possible: Items 6, 8, and 9 with a “yes” answer

**Result:** Apt for occlusion to decannulate: Yes () No ()

Cannula Occlusion: Date: Hour: **Note:** Standardize occlusion with a syringe plunger rubber.

**ASSESSMENT OF THE APTITUDE FOR DECANNULATION**

Apt to attempt decannulation: () Yes = The patient tolerate occlusion for at least 24 hours, with no disocclusion () No = The patient did not tolerate occlusion for 24 hours - Reason:

**Record:** Any occasional need for “disocclusion” for direct tracheal aspiration and how many aspirations were needed:

Decannulated: Yes () Date: / / Name of the professional: No () Reason:

**OBJECTIVE EXAMINATIONS**

Was there an indication for bronchoscopy: No () Yes ()

**Result:** Do the bronchoscopy findings allow decannulation?

Was there an indication for fiberoptic nasolaryngoscopy: No () Yes ()

**Result**: Do the nasolaryngoscopy findings allow decannulation?

Was there an indication for videofluoroscopy? () No () Yes

Result:

**Decannulated:** Yes () No () Reason:

Date: / / Signature:
