# Supplementary material for: Content validation of the decannulation protocol for adult tracheostomized patients
Source: Codas. 2023 Sep 8;35(4):e20210266. doi: 10.1590/2317-1782/20232021266en (PMC10546920; doi:10.1590/2317-1782/20232021266en)
Supplement: Translation

# Translation: Validação de conteúdo de protocolo de decanulação de pacientes adultos traqueostomizados

**DOI:** 10.1590/2317-1782/20232021266pt

**Authors:** Margaret Mendonça Diniz da Côrte, Laélia Cristina Caseiro Vicente, Amélia Augusta de Lima Friche

**Affiliations:** 1 Departamento de Fonoaudiologia, Faculdade de Medicina, Universidade Federal de Minas Gerais - Belo Horizonte (MG), Brasil.

**Keywords:** Validação de Dados, Estudo de Validação, Traqueostomia, Fonoaudiólogo, Fisioterapeuta, Médicos

## Abstract

**Objetivo:**

Realizar a validação de conteúdo de um protocolo de decanulação de pacientes adultos traqueostomizados.

**Método:**

Para a validação de conteúdo do protocolo elaborado por fonoaudiólogas foi utilizada a técnica Delphi. Os 11 itens do protocolo foram julgados por peritos, por meio de rodadas via e-mail e foram classificados como adequados, parcialmente adequados ou inadequados, além de fornecerem comentários e sugestões sobre cada item. Foram convidados 30 fonoaudiólogos, 30 fisioterapeutas respiratórios e 30 médicos responsáveis pelo procedimento de traqueostomia e decanulação. O percentual de concordância adotado foi ≥ 80% e o processo foi interrompido quando obtido esse percentual em todos os itens.

**Resultados:**

Ao final do processo, 24 profissionais participaram da terceira rodada, sendo 46% fonoaudiólogos, 29% fisioterapeutas e 25% médicos. Após as sugestões e comentários dos peritos, dois itens foram mantidos como estavam no protocolo inicial, sete foram reformulados, seis incluídos e dois excluídos. A versão final do protocolo incluiu: identificação, ausência de secreções abundantes, características da secreção, tosse eficaz, capacidade para remover secreções, tolera o balonete desinsuflado, aptidão no processo de decanulação, nível de consciência, troca de cânula para menor calibre, ausência de infecção vigente/ ativa, deglutição espontânea e eficaz de saliva, uso de válvula de fala, aptidão à oclusão da cânula, avaliação da aptidão para decanulação e exames objetivos.

**Conclusão:**

Por meio da Técnica Delphi houve a validação do conteúdo do instrumento, ocorrendo mudanças substanciais. A próxima etapa de validação do instrumento é a obtenção de evidências de validade em relação a estrutura interna.

## INTRODUÇÃO

No ambiente hospitalar a traqueostomia é um procedimento cirúrgico de rotina, que consiste na inserção de uma cânula através de um orifício na traqueia, comunicando-a com o meio externo e tornando a via aérea pérvia^(1)^.

Na última década, evidencia-se aumento no número de traqueostomias realizadas em nível hospitalar^(1,2)^, estimando-se que entre 10 a 15% dos pacientes em unidade de terapia intensiva submetidos à ventilação mecânica necessitarão de traqueostomia como parte de seus cuidados, além das traqueostomias feitas por várias especialidades cirúrgicas fora dos cuidados intensivos^(2,3)^.

Várias são as indicações para a realização do procedimento de traqueostomia e, quando não há mais a necessidade de se manter uma via aérea artificial, ocorre o processo de retirada, denominado decanulação^(2)^, que é uma etapa essencial para a evolução clínica e reabilitação do paciente traqueostomizado não mais dependente da ventilação mecânica^(4)^, porém, as evidências científicas sobre decanulação são limitadas e ainda não há recomendações padronizadas ou protocolos validados para o procedimento^(2,3)^.

Os dados encontrados na literatura sobre a aptidão/prontidão para decanulação foram limitados à opinião de especialistas, estudos de pesquisa, experiência em um único centro, pontuações não validadas para prever o sucesso da decanulação e alguns ensaios clínicos randomizados que se concentraram em questões organizacionais, como equipes de traqueostomia conduzidas por intensivistas ou os efeitos de decisões específicas em desfechos como disfagia ou qualidade do sono^(2-7)^, onde o processo de decanulação é mais frequentemente individualizado do que protocolizado^(6,7)^.

Não há, no nosso conhecimento, protocolo validado para guiar o processo de decanulação, sendo encontrados na literatura alguns artigos sobre as modificações fisiológicas ocorridas após a decanulação, com opiniões divergentes entre os especialistas sobre o tema^(3,6,7)^.

A validação de um protocolo pode ser entendida como um procedimento metodológico pelo qual é avaliada sua qualidade, que pode ser definida como a capacidade de um protocolo medir com precisão o que pretende medir, ou seja, o fenômeno estudado^(6-8)^. A validade de conteúdo é a determinação da representatividade de itens que expressam um conteúdo, baseada no julgamento de especialistas em uma área específica^(6-8)^.

A validação de conteúdo possibilita associar conceitos abstratos com indicadores observáveis e mensuráveis contidos em um instrumento de avaliação, determinando sua representatividade e evidenciando se este explora, de maneira efetiva, os quesitos para a mensuração de um determinado fenômeno a ser investigado, por meio de alguma estratégia metodológica escolhida para tal^(1,2,9-13)^.

A técnica Delphi é amplamente utilizada em estudos de validação de conteúdo, compondo a metodologia de diversas áreas e abordagens^(9-12)^, sendo descrito na literatura um estudo que utilizou a referida técnica e que obteve consenso de especialistas em uma lista de pré-requisitos a serem considerados para a decanulação de traqueostomia em adultos: cura ou reversão do quadro clínico que indicou a realização da traqueostomia, tolera oclusão da cânula de traqueostomia sem estridor, patência das vias aéreas adequada (avaliada pela laringoscopia), adequado nível de consciência, funções laringofaringeas de proteção de vias aéreas preservadas (tosse, deglutição de saliva, capacidade de mobilizar e eliminar secreções), presença de tosse eficaz e ausência de novas propostas cirúrgicas e anestésicas^(10)^. No referido estudo, os autores recomendam fortemente a consideração adicional de alguns parâmetros, tais como o tipo e quantidade de secreções e frequência de aspiração necessária^(10)^. Sendo assim, no presente estudo, julgou-se necessário realizar nova abordagem das indicações à luz da técnica Delphi, para analisar as variáveis em uma perspectiva mais atualizada e adequada à realidade pesquisada, inclusive, incluindo pré-requisitos que não se encontravam presentes no estudo descrito pelos autores no estudo citado.

A multiplicidade de perspectivas associada ao grupo de especialistas produz um resultado mais válido do que um julgamento dado por um especialista individual, mesmo que esse especialista seja o melhor em sua área^(1,9,12)^.

É importante ressaltar que os instrumentos de avaliação e protocolos clínicos são partes integrantes da prática clínica, da avaliação em saúde e de pesquisas, capazes de apresentar resultados cientificamente robustos quando são desenvolvidos e validados de maneira apropriada^(7,14-16)^ sendo que a submissão de seu conteúdo à avaliação de especialistas permite refinar o instrumento para realização posterior dos procedimentos de validação e confiabilidade.

Diante do exposto, o presente artigo tem como objetivo realizar a validação de conteúdo de um protocolo multidisciplinar da decanulação de pacientes adultos traqueostomizados por meio da Técnica Delphi.

## MÉTODO

O estudo obedeceu aos Critérios da Ética em Pesquisa com Seres Humanos, Resolução 466/2012 do Conselho Nacional de Saúde, sendo aprovado pelo Comitê de Ética em Pesquisa (CEP) da instituição, com número de aprovação 4.458.519 e os participantes assinaram Termo de Consentimento Livre e Esclarecido, consentindo, desta forma, com a realização e divulgação da pesquisa e dos seus resultados.

A primeira versão do protocolo de decanulação foi elaborada inicialmente a partir de revisão de literatura nacional e internacional acerca dos critérios de decanulação e de dados dos prontuários de 189 pacientes adultos traqueostomizados hospitalizados, coletados e submetidos a tratamento estatístico^(17)^. Nesse estudo, foi realizado estudo metodológico, quanti-qualitativo de validação de protocolo de decanulação de traqueostomia de uso temporário.

Os itens do protocolo inicial contemplam as variáveis com significância estatística no referido estudo, acrescidas de itens considerados relevantes na literatura da área^(3-7,17)^. Assim, a primeira versão do protocolo incluiu as seguintes itens: capacidade de remover secreções deglutindo ou cuspindo; ausência de secreções abundantes, com necessidade de aspiração traqueal com sonda no máximo três vezes em cada 08 horas e tolerância à oclusão da cânula de traqueostomia num período mínimo de 48 horas; nível de consciência com pontuação entre 12 a 15, de acordo com a Escala de coma de Glasgow (ECG); ausência de infecções ativas; presença de deglutição espontânea de saliva; resultado negativo do Teste *Blue Dye*; tolerância ao balonete desinsuflado permanentemente por no mínimo 24 horas; realização de troca de cânula plástica por metálica; ausência de disfagia; dieta por via oral liberada para as refeições e uso de válvula de fonação.

Para a validação de conteúdo da primeira versão do protocolo de decanulação em adultos, foi utilizada a técnica Delphi, a fim de coletar opiniões de peritos no assunto, tabular os dados e avaliar os critérios para o procedimento.

A técnica Delphi tem seu nome derivado do Oráculo de Delfos, lugar procurado pelos gregos antigos para obtenção de conselhos e respostas sobre o futuro^(9)^. É uma estratégia metodológica de pesquisa e de validação de instrumentos através do consenso de opiniões de um grupo de especialistas por meio de questionários estruturados articulados em fases, ciclos, rodadas ou *rounds* ^(18-20)^, visando obter um máximo de consenso de um grupo de especialistas sobre um determinado tema, quando a unanimidade de opinião não existe em virtude da falta de evidências científicas ou quando há informações contraditórias^(8,21-23)^.

Os especialistas foram selecionados pelos pesquisadores com base no conhecimento e experiência sobre o tema pesquisado e foram convidados a fornecer opiniões sobre este assunto específico, por meio do preenchimento de um questionário avaliativo. Os questionários foram preenchidos de forma anônima entre o grupo^(5,6,21,23)^.

Os resultados foram analisados pelos pesquisadores entre cada rodada de questionários. Foram observadas as tendências e as opiniões dissonantes, bem como suas justificativas, sistematizando-as e compilando-as para, posteriormente, as reenviar ao grupo. Assim, depois de conhecer as opiniões dos outros membros e a resposta do grupo, os participantes tiveram a oportunidade de refinar, alterar ou defender as suas respostas e enviar novamente aos pesquisadores, para que eles reelaborassem o novo questionário a partir dessas novas informações. Esse processo foi repetido até se atingir um consenso^(24)^.

Para compor a amostra do estudo foi definida a participação de pelo menos 30 especialistas peritos no assunto, com especialização e experiência prática/clínica mínima de cinco anos em decanulação de pacientes traqueostomizados e com formações distintas (10 médicos responsáveis pelo procedimento de traqueostomia e decanulação, 10 fisioterapeutas respiratórios e 10 fonoaudiólogos).

Como no transcorrer do método Delphi, espera-se índice de abstenção de 30 a 50% na primeira rodada e de 20 a 30% na segunda rodada^(24)^, foram convidados 90 participantes (30 fonoaudiólogos, 30 fisioterapeutas respiratórios e 30 médicos responsáveis pelo procedimento de traqueostomia e decanulação).

O convite aos especialistas foi feito por via eletrônica, e-mail, mediante carta formal contendo os objetivos, finalidade e desenvolvimento do estudo, etapas, tempo estimado, prazos para devolução das respostas aos questionários e demais detalhes inerentes ao estudo. Anexo ao e-mail foram enviados o manual explicativo do protocolo e o link para acesso ao questionário online avaliativo do protocolo.

Todos os itens do protocolo inicial, bem como dos protocolos reformulados de acordo com a sugestão dos especialistas e reenviados para apreciação, foram avaliados e pontuados por eles de acordo com a escala Likert, em adequado(3), parcialmente adequado(2) ou inadequado(1).

As observações, comentários e sugestões dos especialistas foram registradas em planilha do Excel, relacionadas a cada item avaliado, para posterior análise e modificações sugeridas.

A cada rodada foi avaliada a concordância entre as avaliações dos especialistas pelo Índice de Validação de Conteúdo (IVC)^(11,12,25)^, calculado pelo número de avaliadores concordantes com o item dividido pelo número total de avaliadores. As observações e sugestões dos especialistas foram registradas em arquivo separado e utilizadas, a cada rodada, para reformulação e ajuste dos itens do protocolo.

O percentual de concordância adotado a cada rodada para seleção das variáveis consideradas pertinentes ao protocolo, escolhido de acordo com as indicações da literatura, foi valor maior ou igual a 80%^(6,19)^. Os itens foram mantidos quando o IVC (Índice de Validação de Conteúdo) foi ≥ 80% e revisados quando havia sugestões para reformulação. Foram excluídos quando IVC foi ≤ 80%. Após os ajustes sugeridos pelos especialistas, o protocolo era reenviado para nova apreciação dos especialistas. O processo foi interrompido quando o percentual de concordância foi obtido com relação a todos os itens do protocolo.

Os participantes foram caracterizados em relação à profissão/ocupação e às características sociodemográficas, como idade, sexo, profissão, tempo de conclusão do curso de graduação, pós-graduação e tempo de experiência com pacientes traqueostomizados. Para tal, realizou-se análise descritiva por meio de frequência absoluta e relativa das variáveis.

## RESULTADOS

Para a validação de conteúdo do protocolo, foram necessárias três rodadas até que a concordância dos especialistas participantes sobre todos os itens do protocolo atingisse o percentual estabelecido de 80% (Figura 1).

**Figura 1:** Esquema das 3 rodadas da Técnica Delphi

Na primeira rodada, o questionário foi respondido por 39 dos 90 especialistas convidados, sendo 19 fonoaudiólogos, 11 médicos e 09 fisioterapeutas, portanto, com índice de abstenção de 57% entre o convite e a participação na primeira rodada da técnica. Nas rodadas seguintes, o índice de abstenção observado foi de 26% na segunda rodada (29 dos 39 especialistas responderam ao segundo questionário) e 17% na terceira e última rodada (24 dos 29 especialistas responderam).

Nas três rodadas de avaliação realizadas nesse estudo, participaram profissionais de 28 a 52 anos, com média de idade de 40 anos (dp=6). A maior parte do grupo de especialistas participantes foi de fonoaudiólogos tanto na primeira rodada (49%) quanto na segunda (48%) e na terceira (46%), seguida dos médicos (28%) e dos fisioterapeutas (23%) na primeira rodada. Na segunda e terceira rodadas, as respostas foram enviadas por fonoaudiólogos (45,8%), seguidos pelos fisioterapeutas (29,2%) e médicos (25%) (Tabela 1).

**Tabela 1:** Variáveis sociodemográficas e profissionais dos juízes participantes da Técnica Delphi

**Variável**	% ou média*	
	**Primeira rodada (n=39)**	Última rodada** **(n=24)**
**Idade** (28 a 52 anos)	40*	40* dp=6***
**Sexo**		
Feminino	52	58
Masculino	48	42
**Profissão**		
Fonoaudiólogo	49	46
Médico	28	25
Fisioterapeuta	23	29
**Tempo de conclusão do curso de graduação**		
06 a 10 anos	18	21
11 a 20 anos	59	54
21 a 30 anos	23	25
**Pós-graduação**		
Especialização	100	100
Mestrado	67	29
Doutorado	62	75
Pós doutorado	28	17
**Tempo de experiência com pacientes traqueostomizados**		
Até 10 anos	31	38
De 11 a 20 anos	54	54
Mais de 20 anos	15	8

* Valor apresentado em média **Terceira rodada ***Desvio padrão

Com relação aos dados profissionais, a maioria dos participantes tem mais de 11 anos de formado (75%) e todos (100%) possuem Especialização na área de atuação. Na primeira rodada, a proporção entre os que possuem mestrado (67%) e doutorado (62%) são semelhantes, se diferenciando dos resultados dos que possuem pós-doutorado (28%). Na terceira e última rodada, com relação ao número de profissionais que possuem mestrado (29%) e doutorado (75%), foi observada uma diferença importante. O tempo de experiência com pacientes traqueostomizados predominante entre os especialistas participantes foi entre 11 a 20 anos (Tabela 1).

Após esta rodada, todos os itens tiveram índice de concordância ≥ 80% finalizando, então, a técnica Delphi, sendo o protocolo considerado validado, no que se refere ao seu conteúdo. Os itens do protocolo avaliados pelos especialistas encontram-se descritos abaixo (Tabela 2).

**Tabela 2:** Itens avaliados nas 3 rodadas da Técnica Delphi

Itens avaliados	Rodada/IVC (%)		
	1ª	2ª	3ª
Dados de Identificação	38*	79*	96
Dados Complementares	**	96	96
Aptidão para início do Processo de Decanulação			
Ausência de secreções abundantes	59*	90	90
Quantidade de secreção a cada aspiração	**	76*	88
Aspecto da secreção	**	82	88
Capacidade para remover secreções	85*	97	97
Tosse eficaz	64*	90	90
Tolera o balonete desinsuflado por 24 hs	**	86	86
**Resultado:** Apto a iniciar processo	79*	80	89
Aptidão à Oclusão da Cânula de Traqueostomia			
Nivel de cs.: ECG 09 a 12/13 a l5	59*	83	83
Troca para cânula metálica de menor calibre	**	83	90
Ausência de infecções ativas	59*	90	90
Presença de deglutição espontânea de saliva	95	93	93
Teste Blue Dye	34*	***	***
Dieta por VO liberada	59*	***	***
**Resultado:** Apto à oclusão	66*	83	83
Aptidão para a Decanulação			
Data/hora da oclusão; Êmbolo, Gaze, Esparadrapo	69*	***	***
Oclusão da cânula: Data/hora; Padrão: êmbolo	**	86	86
Oclusão (hs): 24-36/48-72/Não tolera	79*	83	83
Broncoscopia: N/S; Data e resultado	54*	***	***
Exames objetivos: Bronco/Fibronaso/Videodegluto	**	93	93
**RESULTADO**			
Apto à decanulação	74***	***	***
Apto: Tolerou 24 h. Se exames: Não contraindicam	**	80*	92
Decanulado(a): Sim/Não; Motivo	97	97	96
Data/hora da decanulação e nome do profissional	97	93	96

* Reformulado **Incluído ***Excluído

**Legenda:** IVC = Índice de validação conteúdo

Os itens foram mantidos tal como estavam quando o IVC (Índice de Validação de Conteúdo) foi ≥ 80% e não havia sugestões de modificação por nenhum dos especialistas, caso contrário, foram reformulados e reenviados para avaliação na próxima rodada. De acordo com as sugestões e comentários dos peritos, dois itens foram mantidos como estavam no protocolo inicial, sete foram reformulados, seis incluídos e dois excluídos. O protocolo final, após a terceira rodada, pode ser consultado no Apêndice A desse artigo.

De acordo com as sugestões e comentários dos peritos, os itens capacidade para remover secreções deglutindo ou cuspindo e presença de deglutição espontânea de saliva foram mantidos como estavam no protocolo inicial.

Os sete itens reformulados são apresentados a seguir, acompanhados dos principais comentários/sugestões dos especialistas: dados de identificação (a identificação do paciente participante do estudo deveria ser feita somente com as iniciais, para preservar a identidade do mesmo; acrescentar data da internação hospitalar e peso e altura), ausência de secreções abundantes (anotar quantidade de vezes que necessita ser aspirado na prática, pois faz diferença ter zero aspiração e 3 aspirações; é fundamental quantificar a secreção aspirada; pois é necessário, para proteção de vias aéreas, conseguir eliminar as secreções), tosse (nem todos os serviços dispõem do medidor de fluxo de tosse), nível de consciência (limitar o resultado a de 9 a 15 na escala de coma de Glasgow, por ser um perfil de pacientes mais indicado à decanulação), ausência de infecção ativa (especificar infecções ativas pulmonares ou larigofaríngeas, pois, alterações pulmonares e neurológicas, por exemplo, podem interferir no processo de decanulação); oclusão da cânula e avaliação da aptidão para decanulação.

Os seis itens incluídos foram dados complementares, características da secreção, tolera o balonete desinsuflado, troca de cânula por menor calibre, uso de válvula de fala e exames objetivos realizados.

Foi sugerida a exclusão dos itens teste Blue Dye e dieta por via oral liberada. No que se refere ao teste Blue Dye, foi sugerida a exclusão, considerando-se que o teste pode ser falso negativo em até 50% dos casos, não sendo um parâmetro confiável para ser considerado, além de ser a avaliação clínica fonoaudiológica considerada suprema em relação ao teste. Com relação ao item dieta por VO liberada, as principais observações dos especialistas foram que nem sempre a possibilidade de decanulação do paciente será relacionada à condição de receber dieta por via oral (VO), pois é um parâmetro que analisado sozinho não indica critérios e riscos para decanulação de traqueostomia, portanto, não há relação e dependência direta da possibilidade de liberação de dieta por VO com a decanulação. Além disto, pacientes disfágicos, com impossibilidade de liberação de dieta por VO podem ter uma via aérea pérvia e capacidade de proteção de vias aéreas inferiores que permita a decanulação.

O protocolo final, após a terceira rodada, pode ser consultado no Apêndice A desse artigo.

Por fim, de acordo com observação comparativa entre os conteúdos dos protocolos inicial e final, observamos que as principais modificações realizadas, de acordo com a avaliação e observações dos especialistas foram: entre a primeira e a segunda rodadas, a inclusão dos itens protocolo interdisciplinar; doenças respiratórias e disfagia pregressas; motivo da realização da TQT e intercorrências; diâmetro da cânula de traqueostomia e ausência ou presença de Cuff, características de viscosidade e aspecto da secreção, uso de válvula de fonação e tolerância ao balonete desinsuflado e a modificação/reformulação dos itens ausência de secreções abundantes e tosse.

As mudanças observadas entre a segunda e a terceira rodadas foram relacionadas à quantidade e aspecto da secreção aspirada e avaliação da tosse.

Após as três rodadas com as sugestões dos especialistas, os itens que permaneceram na versão final do protocolo foram: identificação, ausência de secreções abundantes, características da secreção, tosse eficaz, capacidade para remover secreções, tolera o balonete desinsuflado, critérios: aptidão ao processo de decanulação, nível de consciência (ECG), troca de cânula para menor calibre, ausência de infecção vigente/ ativa, deglutição espontânea e eficaz de saliva, uso de válvula de fala (VF), critérios: aptidão à oclusão da cânula, avaliação da aptidão para decanulação, exames objetivos (Apêndice A).

## DISCUSSÃO

As três categorias profissionais participantes são especialidades intimamente envolvidas no processo de decanulação e cujas decisões são determinantes da condução e do desfecho de todo o processo, o que possibilitou a construção mais confiável e sem a ocorrência de dominância individual do instrumento de avaliação proposto nesse estudo^(1)^.

A falta de protocolo validado de decanulação em serviços hospitalares pode ocasionar complicações clínicas e respiratórias, como decanulação prematura, insuficiência respiratória, acúmulo de secreções em recessos faríngeos com aumento de risco de broncoaspiração, comprometimento do mecanismo de proteção das vias aéreas inferiores, estridor em vias aéreas inferiores, sepses, aumento do estoma e alterações de mucosa^(26)^.

A validação de protocolos em saúde se constitui em tarefa importante quando se pretende possibilitar segurança, evidências e qualidade às ações relacionadas à assistência ao paciente, especialmente no contexto hospitalar, no sentido de promover ações seguras, eficazes e eficientes^(23-26)^.

A técnica Delphi é utilizada com o objetivo de gerar uma amostragem da opinião de especialistas, evitando a dominância por indivíduos particularmente assertivos. Assim, tem sido considerada como uma forma adequada de extrair dados úteis de experiências pessoais que podem ser transformadas em dados empíricos para estudos futuros^(7-9)^. Com base nas respostas das técnicas Delphi, são elaboradas diretrizes que atuam como uma base importante para a realização e avaliação de estudos ou publicações^(1,9)^.

As evidências de relatórios ou opiniões de comitês de especialistas e / ou experiência clínica de autoridades respeitadas se enquadram no Nível IV da pirâmide de evidências^(22)^. A maioria dos especialistas concorda que quanto mais alto na hierarquia estiver posicionado o desenho do estudo, mais rigorosa será a metodologia^(22)^.

Os especialistas participantes do estudo apresentaram satisfatório perfil de experiência clínica na área, com muitos anos de experiência com pacientes traqueostomizados, o que corrobora a literatura, que preconiza que os peritos participantes deverão apresentar afinidade com a proposição que se busca validar, portanto, devem possuir produção acadêmica, científica e/ou experiência profissional na área em que se fundamenta o estudo de maneira que o configure como um perito^(23)^.

Com relação à formação dos especialistas, todos possuíam pós-graduação em nível de especialização, uma amostra numericamente expressiva possuía mestrado e doutorado. A literatura refere que, para se obter uma avaliação com boa qualidade, é recomendado selecionar um painel de especialistas qualificados no tema, com características de formação acadêmica e *expertise* de acordo com a questão a ser analisada, devendo tomar por base a qualidade de sua contribuição^(10,23)^. A expertise está relacionada a um *continuum*, que inclui expertise subjetiva e expertise objetiva, ambas relacionadas à formação acadêmica e à experiência relacionada ao tema pesquisado^(23)^. Desta forma, os especialistas devem ser escolhidos pela sua experiência e credibilidade no assunto^(25,26)^, sendo sugerido um tamanho de amostra de no mínimo 10 (um tamanho menor não gera ideias suficientes) e de no máximo 50 participantes (uma amostra maior resulta em ineficiências de custo relacionadas a tempo, produto e processo de iteração) especialistas peritos no assunto, com diferentes formações, para ampliar o raciocínio clínico em torno do assunto investigado^(25-28)^.

No presente estudo, apesar da proposta inicial ter a perspectiva de contar com a participação de um mínimo de 30 especialistas, observou-se um número final de 24 participantes na terceira rodada com bom índice de validação do conteúdo. Ademais, apesar do número final de especialistas ter sido menor do que o esperado, constata-se que este número se encontra dentro do sugerido na literatura como adequado para manter a qualidade da Técnica Delphi no consenso de opiniões^(1,24,27,29)^.

As abstenções não alteraram a qualidade do processo de validação do conteúdo do protocolo de decanulação. Considera-se que a falta homogeneidade das categorias profissionais em todas as rodadas pode ter sido uma fragilidade relacionada ao estudo, já que se pretende um protocolo multiprofissional.

Por outro lado, a análise do perfil dos profissionais demonstrou que os participantes têm formação e tempo de experiência adequados na área de decanulação de pacientes traqueostomizados, o que possibilita análises, observações e sugestões adequadas e pertinentes ao tema.

Assim, de acordo com as observações e sugestões dos especialistas, as principais alterações ocorridas na versão 1 do protocolo se referem aos itens dados complementares (especialistas sugerem inclusão de alguns itens após a identificação, a fim de incorporar detalhes sobre o caso clínico), quantificação e identificação das características das secreções, tolerância à deflação do balonete, uso de válvula de fala, padronização do recurso para oclusão da cânula e retirada dos itens teste *Blue Dye* e dieta por via oral liberada. Tais alterações culminaram na versão 2, que, por sua vez, teve como principais sugestões de modificação os itens aspectos detalhados das secreções, uso de válvula de fala, exames objetivos realizados e critérios de aptidão à decanulação. As modificações decorrentes desta segunda rodada, culminaram na versão 3 do protocolo, que pode ser consultada no Apêndice A.

Com relação ao item dieta por VO liberada um percentual importante de especialistas médicos, fisioterapeutas e fonoaudiólogos, questionou a relação direta entre a habilidade do paciente para a liberação e ingesta de dieta por via oral e a aptidão para a decanulação. Não foram encontrados estudos que abordaram a relação entre decanulação e a liberação da dieta por VO.

Dentre as considerações dos especialistas, observou-se sugestão de exclusão ou de desconsideração deste item como importante parte do protocolo pelos especialistas, sendo a maior parte destes, fonoaudiólogos e, considerando-se que “o fonoaudiólogo é o profissional legalmente habilitado para realizar a avaliação, diagnóstico e tratamento fonoaudiológicos das disfagias orofaríngeas, bem como o gerenciamento destas no recém-nascido, na criança, no adolescente, no adulto e no idoso” (RESOLUÇÃO CFFa Nº 356, de 06 de dezembro de 2008), optou-se por excluir este item do protocolo.

O protocolo de decanulação proposto e avaliado em um estudo^(16)^ foi elaborado por meio de levantamento na literatura de dados importantes no processo de decanulação considerando-se critérios clínicos e estatísticos, sendo substancialmente modificado e adequado, em termos de conteúdo, conforme avaliação dos especialistas. Assim, os autores consideraram o protocolo validado no que se refere ao seu conteúdo. Conforme descrito na literatura, a validade de conteúdo é a determinação da representatividade de itens que expressam um conteúdo, baseada no julgamento de especialistas em uma área específica, determinando se o conteúdo de um protocolo explora, de maneira efetiva, os quesitos para a mensuração de um determinado fenômeno a ser investigado^(1,2)^, processo que ocorreu neste estudo de validação.

Apesar das possíveis limitações citadas anteriormente, esse estudo é, no nosso conhecimento, o primeiro a propor a validação de protocolo de decanulação em uma perspectiva multiprofissional. O protocolo final contemplou os itens mais importantes para o processo de decanulação, favorecendo a identificação de características clínicas e respiratórias e, consequentemente, a tomada de decisões acertadas para prevenir complicações durante esse processo.

Etapas futuras de validação e de confiabilidade do protocolo devem ocorrer em diferentes serviços com aplicação em pacientes hospitalizados.

## CONCLUSÃO

O presente estudo descreve a validação de conteúdo de um protocolo multidisciplinar de decanulação de pacientes adultos traqueostomizados por meio da Técnica Delphi e, diante dos resultados encontrados, considera-se que as evidências de validade obtidas são satisfatórias.

As contribuições dos especialistas permitiram aprimorar o instrumento, sendo validado o conteúdo. A próxima etapa de sua validação é a obtenção de evidências de validade em relação a estrutura interna, para depois o instrumento ser submetido para obtenção de outros parâmetros de validação e confiabilidade, por meio da aplicação do protocolo com a população alvo.

## Apêndice A Protocolo final

**Protocolo de Decanulação de traqueostomia**

Orientação: Protocolo interdisciplinar: médico, fisioterapeuta e fonoaudiólogo. As avaliações e decisões sobre o processo de decanulação devem ser realizadas pela equipe.

Data da avaliação: Profissional responsável:

Dados de Identificação:

Nome (Apenas identificação numérica à coleta de dados nos hospitais):

DN: Idade: anos Sexo: F () M () Peso: Kg Altura: metros

Dados Complementares à Avaliação Inicial:

Data da Internação: Diagnóstico à internação:

Comorbidades/Doenças respiratórias pregressas/Disfagia prévia:

Realização da TQT: Data: Motivo:

Intercorrências no procedimento: Não () Sim () Quais:

Cânula: Plástica/Silicone: com endocânula () sem endocânula () Metálica ()

Cuff: Ausente () Insuflado: Sim () Não () Diâmetro (número) da cânula:

**APTIDÃO PARA INÍCIO DO PROCESSO DE DECANULAÇÃO**

1. Ausência de secreções abundantes: ≤ 3 aspirações com sonda em 24 hs: Sim () Não () **Orientações:** a) considerar número de aspirações com sonda por dia (24 hs) pelas equipes de: enfermagem, fisioterapia e fonoaudiologia

b) considerar quantidade de secreção aspirada (em geral nas aspirações): Pequena: secreção atingiu apenas a sonda de aspiração; Moderada: atingiu o início da extensão do vácuo; Grande: atingiu o frasco de aspiração

2. Características da secreção:

a) Viscosidade: Fluida () Espessa ()

b) Aspecto: Hialina: clara, transparente () Purulenta () Mucopurulenta () Sanguinolenta () Piosanguinolenta ()

3. Tosse (espontânea e/ou voluntária) eficaz para mobilização de secreção de Vias Aéreas: Sim () Não ()

4. Capacidade para remover secreções deglutindo ou cuspindo: Sim () Não ()

5. Tolera o balonete desinsuflado por 24 hs: Sim () Não ()

Obs: mantém-se estável clinicamente e com padrão respiratório adequado com o balonete permanentemente desinsuflado durante as 24 horas do dia

Critérios para aptidão ao início do processo de decanulação:

Todos os ítens resposta “Sim” e item 3 respostas: fluida e hialina

**Resultado:** Apto a iniciar processo de decanulação: Sim () Não ()

**APTIDÃO PARA OCLUSÃO DA CÂNULA DE TRAQUEOSTOMIA**

6. Nível de consciência (Escala de Coma de Glasgow) com pontuação 9 a 15: Sim () Não ()

7. Troca de cânula para menor calibre: Sim () Não ()

8. Ausência de infecção vigente/ativa (Critérios: antibioticoterapia, presença de leucocitose): Sim () Não ()

**Considerar:** Infecções ativas pulmonares ou laringofaríngeas; Sepse; Infecções com presença de delirium

9. Presença de deglutição espontânea e eficaz de saliva: Sim () Não ()

10. Uso de válvula de fala (VF): Sim () Não () **Obs:** estável clinicamente e com padrão respiratório adequado com o uso constante da VF . Informação complementar, a ser utilizada nos serviços onde há o recurso do uso da VF

Critérios para aptidão à oclusão da cânula:

Ideal: Todos os ítens resposta “Sim” Possível com segurança: Itens 6; 8 e 9 resposta “Sim”

**Resultado:** Apto à oclusão para decanulação: Sim () Não ()

Oclusão da cânula: Data: Hora: **Obs:** Padronizar oclusão com borracha do êmbolo de seringa

**AVALIAÇÃO DA APTIDÃO PARA DECANULAÇÃO**

Apto à tentativa de decanulação: () Sim = Tolerou oclusão mínima de 24 horas, sem desoclusão () Não = Não tolerou oclusão mínima de 24 horas - Motivo:

**Registrar:** eventual necessidade de “desoclusão” para aspiração traqueal direta e quantas aspirações foram necessárias:

Decanulado: Sim () Data: / / Nome do Profissional: Não () Motivo:

**EXAMES OBJETIVOS**

Houve indicação de exame de Broncoscopia: Não () Sim ()

**Resultado:** Os achados broncoscópicos permitem a decanulação?

Houve indicação de exame de Fibronasolaringoscopia: Não () Sim ()

**Resultado**: Os achados da fibronaso permitem a decanulação?

Houve indicação de exame de Videodeglutograma? () Não () Sim

Resultado:

**Decanulado:** Sim () Não () Motivo:

Data: / / Assinatura:
